# Density dependent attributes of fish aggregative behaviour

**DOI:** 10.7717/peerj.6378

**Published:** 2019-02-04

**Authors:** Michaela Holubová, Martin Čech, Mojmír Vašek, Jiří Peterka

**Affiliations:** 1 Institute of Hydrobiology, Biology Centre of the Czech Academy of Sciences, České Budějovice, Czech Republic; 2 Faculty of Science, University of South Bohemia, České Budějovice, Czech Republic; 3 SoWa, Biology Centre of the Czech Academy of Sciences, České Budějovice, Czech Republic

**Keywords:** Open water, Bream, Shoaling, Schooling, Perch, Bleak, Roach, Freshwater fish, Emergent properties, Critical density

## Abstract

Grouping behaviour, as fascinating as it is unclear, has lately drawn the attention of numerous researchers. While most of the authors focused their work on a mechanistic approach to the matter of schooling, this study explores the issue from a population point of view. Present camera observation study on the fish community carried out in the epipelagic habitat of a European temperate reservoir in the Czech Republic explored the relationship between density and aggregative features of predominantly cyprinid fish stock. Results demonstrated that schooling behaviour is triggered by the ‘critical density’ of fish in the habitat. School size as well as counts of schools and proportion of schooling individuals increased with the density of fish. Counts of clusters (observed units in time, including singletons, pairs and schools) and cluster size, on the other hand, showed a slowing tendency to increase. The slower increase implies the tendency of fish for not being frequent but rather to create larger groups. Altogether, our findings suggest that fish density is a triggering factor in the formation of large fish schools. As the tendency of cyprinid species for school formation could be an evolutional advantage responsible for dominance in later succession phases of water bodies, we suggest that more in situ studies should be encouraged for the proper understanding of the ecological interactions that drive the structure of aquatic ecosystems and for ensuring unbiased assessment.

## Introduction

Fish aggregative behaviour has been puzzling people for decades. Schooling behaviour is mainly considered as an antipredator strategy (Pitcher & Parrish, 1993) convenient particularly for species inhabiting the environment with the lack of shelters such as the epipelagic habitat. This habitat often contains an important food source for zooplanktivores = the planktonic crustaceans. In Římov Reservoir (Czech Republic), mainly adult zooplanktivorous fish inhabit the pelagic environment during the daytime (Říha et al., 2015) when they can be highly conspicuous for predators; therefore, it is convenient for them to seek protection via schooling behaviour (Williams, 1964), although the vulnerability to predators differ with respect to species. There is a growing body of evidence that predators prefer preying on aggregations than on individuals (Botham & Krause, 2005). Especially in a marine environment, large aggregations attract numerous predators and, if localised, the overwhelming majority is often consumed (reviewed in Maury, 2017). Still, the existence of schools proves the tendency of organisms to form a patchy distribution and its advantageousness. Being a member of the school brings various other benefits, such as lower risk of being captured (Hamilton, 1971), increase in detection time by predator (Vine, 1971; Ioannou et al., 2011), faster sighting of approaching predator (Godin, Classon & Abrahams, 1988), faster location of quality food resources (Krebs & Davies, 1993), etc. Although the sighting distance by predators increases with the number of individuals in the group, the benefits from the group are still several times higher than the risk of predation; this is true especially for freshwater-system piscivores which manage to handle no more than few, usually only one, prey fish at once (Ruxton & Johnsen, 2016).

The emergence of fish schools is noted to be dependent on the density of conspecific individuals (Okubo, 1986). In natural conditions, however, the absence of conspecifics might enhance the formation of heterospecific schools with visually and ecologically similar species (Ward, Axford & Krause, 2002). The presence of marginal number of individuals is a trigger which drives loser aggregations into dense schools. School size and composition can repeatedly fluctuate within a short time span (Radakov, 1973). Evered and Seghers (cited in Seghers, 1981) noted that various ambient causes as variations in the encounter rate of conspecific individuals or sympatric species (Okubo, 1986; Croft et al., 2003), or the state of hunger within group (Robinson & Pitcher, 1989) can trigger merging or division of groups (Okubo, 1986; Gueron, 1998). Similarly, the presence or absence of predators can affect the duration of schools as well as their cohesion (Tien, Levin & Rubenstein, 2004); especially in the case of heterospecific schools, the duration of school is more likely to be lower (Wolf, 1985).

Research on freshwater fish schooling was rather neglected (Milne, Shuter & Sprules, 2005) mainly due to low economic importance in comparison with marine habitat. Nevertheless, countless laboratory studies (Wright et al., 2003; Hoare et al., 2004; Hensor et al., 2005) and theoretical models (Okubo, 1986; Gueron & Levin, 1995; Gueron, 1998) have been conducted in order to unveil details on fish schools and shoals including density dependence, yet field observations are still sporadic. Moreover, focus organisms are mostly small bodied species or juvenile individuals (Wright et al., 2003; Guillard et al., 2006). Paradoxically, non-field results may bring inaccurate information as noted by Hensor et al. (2005), who compared the shoaling tendency of banded killifish (*Fundulus diaphanous*) in shallow habitat and laboratory with simulation models. They proved that neither models nor laboratory results reflect the actual situation in the field. Artificial environment can affect the behaviour by various stimuli apart the one that is being studied as have been concluded by Reebs (2002), therefore in situ observations are worth pursuing in order to obtain a ‘true picture’.

The goal of this in situ study was to unveil the relationship between fish density and attributes of schooling behaviour in the open water habitat of temperate freshwater reservoir by describing the actual state from acquired video recording data.

## Methods

### Study area

**Figure 1:**
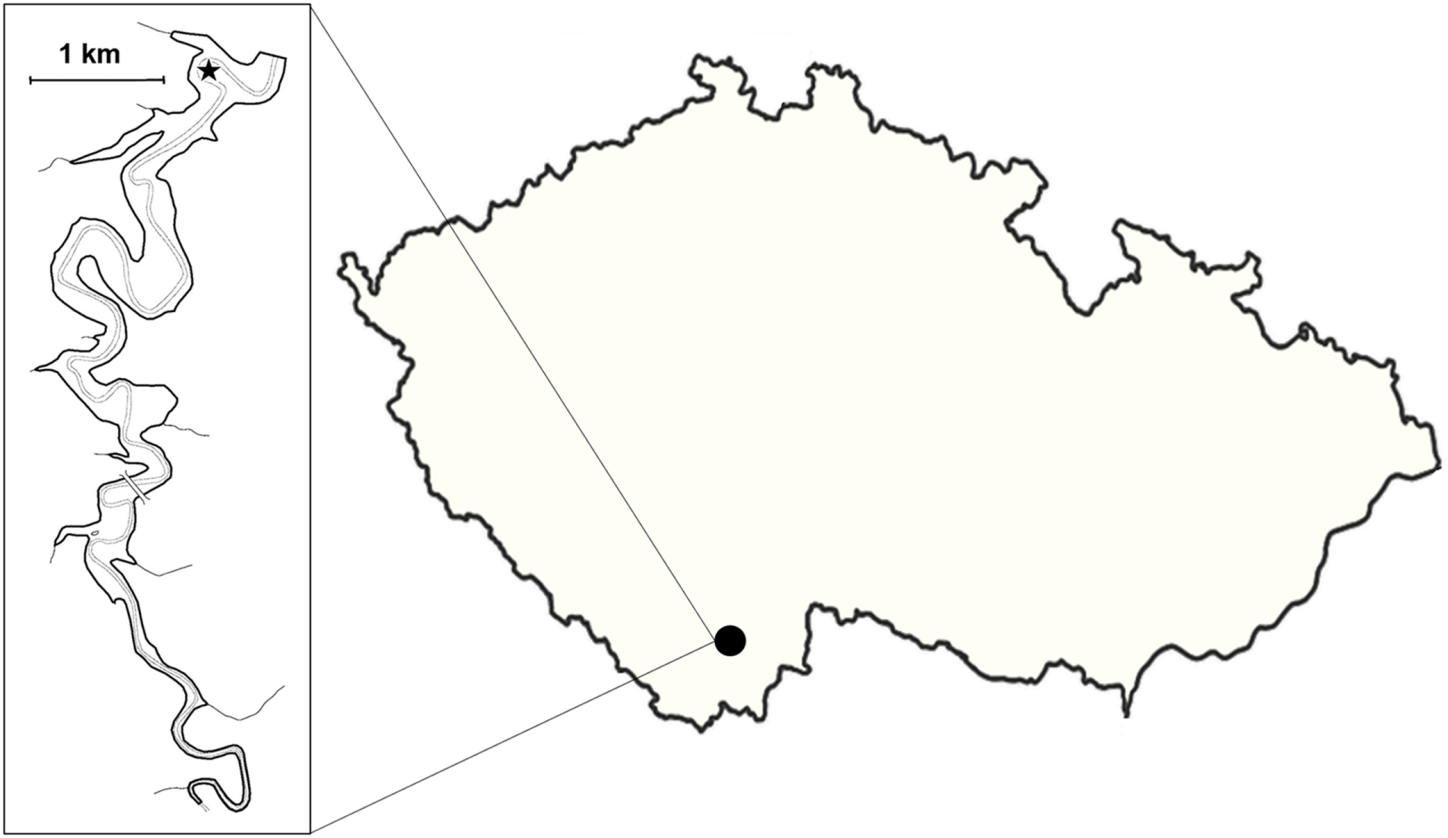
Map of Europe with marked reservoir location. Star is for study site.

The study was conducted in the dam area of canyon-shaped Římov Reservoir built on the Malše River as a drinking water supply for adjacent areas (48.848N, 14.845E; Czech Republic; Fig. 1), therefore no public access is allowed. Researchers of Institute of Hydrobiology are allowed to enter to Římov Reservoir with the permit by Vltava River authority, contract number 300/7225. The total length of the reservoir is approximately 12 km with the max area of 210 ha, volume 33 × 10^6^ m^3^, and maximal surface elevation of 471 m a.s.l. Mean and maximal depth of the reservoir is 16 and 45 m, respectively. Reservoir is dimictic with summer stratification established from April to October. Water transparency (Secchi depth) reaches up to six m during the ‘clear water’ phase whereas summer period transparency is rather low (less than two m). Trophy of the reservoir decreases from eutrophic riverine to mesotrophic dam part (Hejzlar & Vyhnálek, 1998). Cyprinid species dominate the community of the reservoir, namely freshwater bream (*Abramis brama*), roach (*Rutilus rutilus*) and bleak (*Alburnus alburnus*), along with European perch (*Perca fluviatilis)* (Říha et al., 2008). Several predatory species can be found in the pelagic area, specifically asp (*Leuciscus aspius*), wels catfish (*Silurus glanis*), Northern pike (*Esox lucius*), and pike-perch (*Sander lucioperca*) (Prchalová et al., 2008).

Littoral areas are extremely limited particularly in the lower part near to the dam. Steep shores and seasonal water level fluctuation are responsible for deficiency of submerged aquatic macrophytes (Vašek et al., 2009; Čech et al., 2012). The lack of shelters and need for search for food in pelagic zone should be favourable for fish school formation.

### Camera set-up

**Figure 2:**
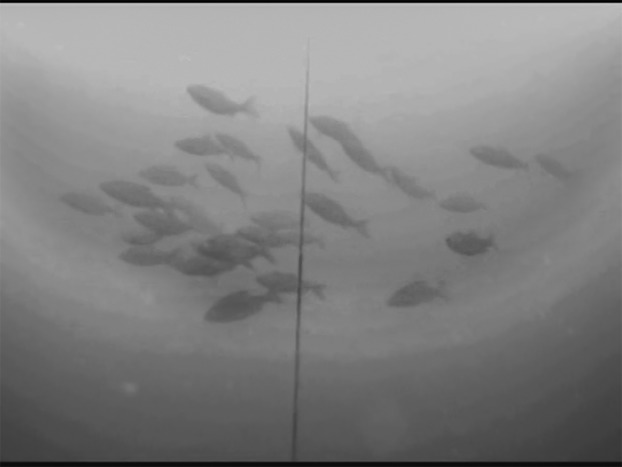
Snapshot from video footage taken by Jiří Peterka, study co-author. School of roach individuals passing above the camera.

**Figure 3:**
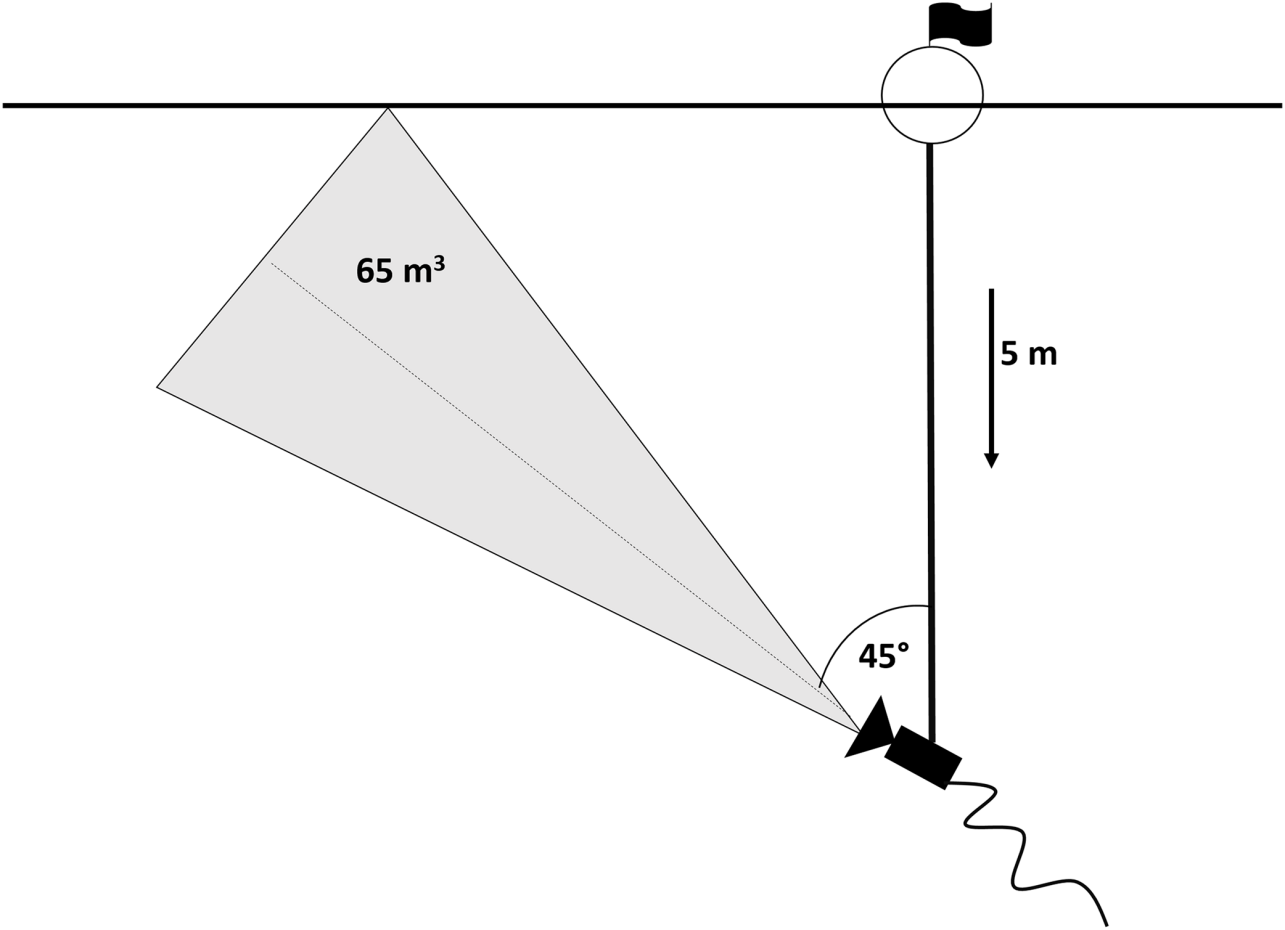
Scheme of the camera set-up.

An underwater video camera (SplashCam Delta Vision HD B/W; OCEAN SYSTEMS, Everett, Washington, USA) used for data recording was mounted on a five m-long metal bar which was attached to a buoy floating on the water surface and secured by two anchors in a fixed position. Previous experiments showed 45° tilt of the camera towards the surface to be the most effective position in order to obtain the highest possible contrast in the visual field (silhouettes of fish positioned against the bright surface; snapshot of actual footage in Fig. 2) (Peterka, Vašek & Matěna, 2006). In this set up, camera took up approximately 65 m^3^ of the epilimnetic layer. The set-up was situated in the pelagic habitat of the dam part, where depth reached 30 m, in the distance of approximately 100 m from reservoir bank. The camera was connected to a computer situated in the floating boat shed for the recordings storage (for scheme see Fig. 3).

### Data collection and processing

The observations were conducted out of the spawning period during the ‘clear water’ phase, May–early June (31 May 2005–5 June 2005, 29 May 2012–5 June 2012, 15 May 2014–31 May 2014, but not all data were used for the analysis, see below). This period being typical for high water transparency even in systems with higher trophy due to grazing activity of planktonic crustaceans (Lampert et al., 1986) is convenient since high transparency is essential for obtaining good quality data using visual census. The recording took place during daytime conditions and 16 h (from 5 AM to 9 PM) of video per day were obtained. During 1 week (May 21–26) in 2014, visual condition were inconvenient and consequently the data excluded from the analysis. The camera output data were captured using the AVS video editor (https://www.avs4you.com/). Recorded files were automatically split and saved every 20 or 60 min and afterwards analysed by means of video editing software Avidemux (http://fixounet.free.fr/avidemux/). Each observation was considered as an independent record unless the repetition of the very same individual was apparent. Some individuals, mostly perch, took interest in camera or cycled around, disappearing and reappearing before camera in very short intervals. If such a repetition occurred, a record in frequency shorter than 2 min was regarded as a repetitive observation (not counted) and an observation in a frequency longer than 2 min was considered as independent one. In total, 263 repeated observations of same individuals were omitted (specifically 204 perch observations, 27 bream, 20 bleak, six roach, five asp and one catfish). Except for several occurrences of fish fry that were not included in this analysis, all observed individuals were considered as adults considering obvious body size and reported prevalence in the epipelagic during daytime (Vašek et al., 2009; Muška et al., 2013). Observed fish were categorised as singletons, pairs or schools. Observed groups of three and more fish were called ‘schools’ because of polarisation and coherence; we did not observed any shoals since we feel that in freshwater habitat shoals are mainly issue of littorals, whereas in pelagic habitat the need for food search pushes fish to form ‘schools’. A pair of fish behaves differently to a school; according to Partridge (1982), there is only leader and follower, whereas in a group of three all fish adjust to each other. Based on this we treated pairs separately. Though in most cases the period between observations of fish was several minutes, coincidental observation of several individuals was recorded as a school if the coherence and polarisation of all individuals in the school was indisputable; otherwise, the individuals were considered independently. Inter individual distance between school members estimated from video recordings, that is, distance between front and tailing individuals, was mostly about length of fish body, but we counted as a school member even fish lagging more than one body length behind the school if they clearly followed school trajectory. Observed schools were distant enough from camera to claim that vast majority of encounters provided recordings of whole schools. All categories (i.e. singletons, pairs and schools) were summed as clusters for analyses of aggregation. With a few exceptions, species were distinguishable in the videorecordings. Because of the presence of heterospecific schools, the data were not separated by species especially since there is an evidence that the species in the heterospecific groups adjust to each other (Tang et al., 2017). Moreover, size of heterospecific group probably undergoes different pressures and might be more easily dismissed (Wolf, 1985). On several occasions technical difficulties during video recording caused loss of video data, therefore data from only 24 days of observation were used (days with more than three missing hours were not included into analysis). Missing hours in these days (6 days with one missing hour, 2 days with two missing hours, and 1 day with one three missing hour) were supplemented with average hourly values to obtain 16 h of observation.

Several models (basic linear level-level, log-level, log-log, exponential growth and logistic grow curve with carrying capacity) were tested to find the best fit for modelling the density dependence of aggregation attributes. For the analyses, the values summarised per day were used in case of fish density, cluster counts, school counts and counts of individuals in size categories (singletons, pairs and schools). In case of school and cluster sizes values were averaged per day.

Best fit for the relationship between cluster size (cluster is observed unit of any size) and fish density was obtained by log-log model, as well as count of schools. Count of clusters and fish density was fitted with log-level model. A linear regression proved best fit for modelling relationships between schools size and fish density, as well as relationship of proportions of fish in size categories (singletons, pairs and schools). Counts of fish in size categories (singletons, pairs and schools) and fish density were fitted with the log-log models. If logarithmic transformation was used in the analysis, the values for graphical presenting were back exponentiated for better understanding of analyses output. In all analyses best fitted models were chosen on the basis of the lowest value of Akaike Information Criterion. For demonstrating ‘critical density’, hourly proportions of fish in categories (singletons, pairs and schools) were used (322 h of observation). Statistical analyses and graphical visualisation were conducted in R project statistical computing software, using packages stats (R Core Team, 2017), ggplot2 (Wickham, 2009) and stargazer (Hlavac, 2018).

## Results

**Figure 4:**
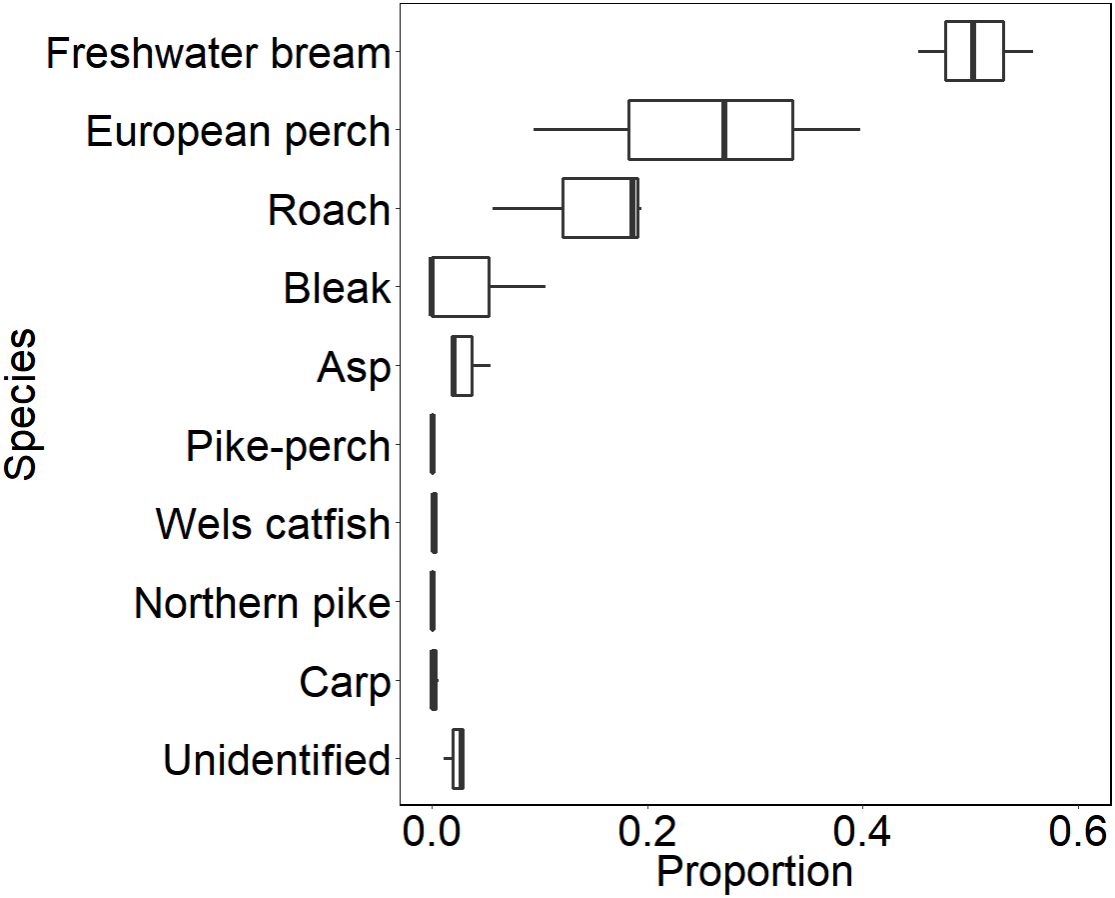
Boxplot showing relative species composition during three-season observation. Median values (thick lines), upper and lower quartiles (boxes), minimum and maximum values (whiskers).

**Figure 5:**
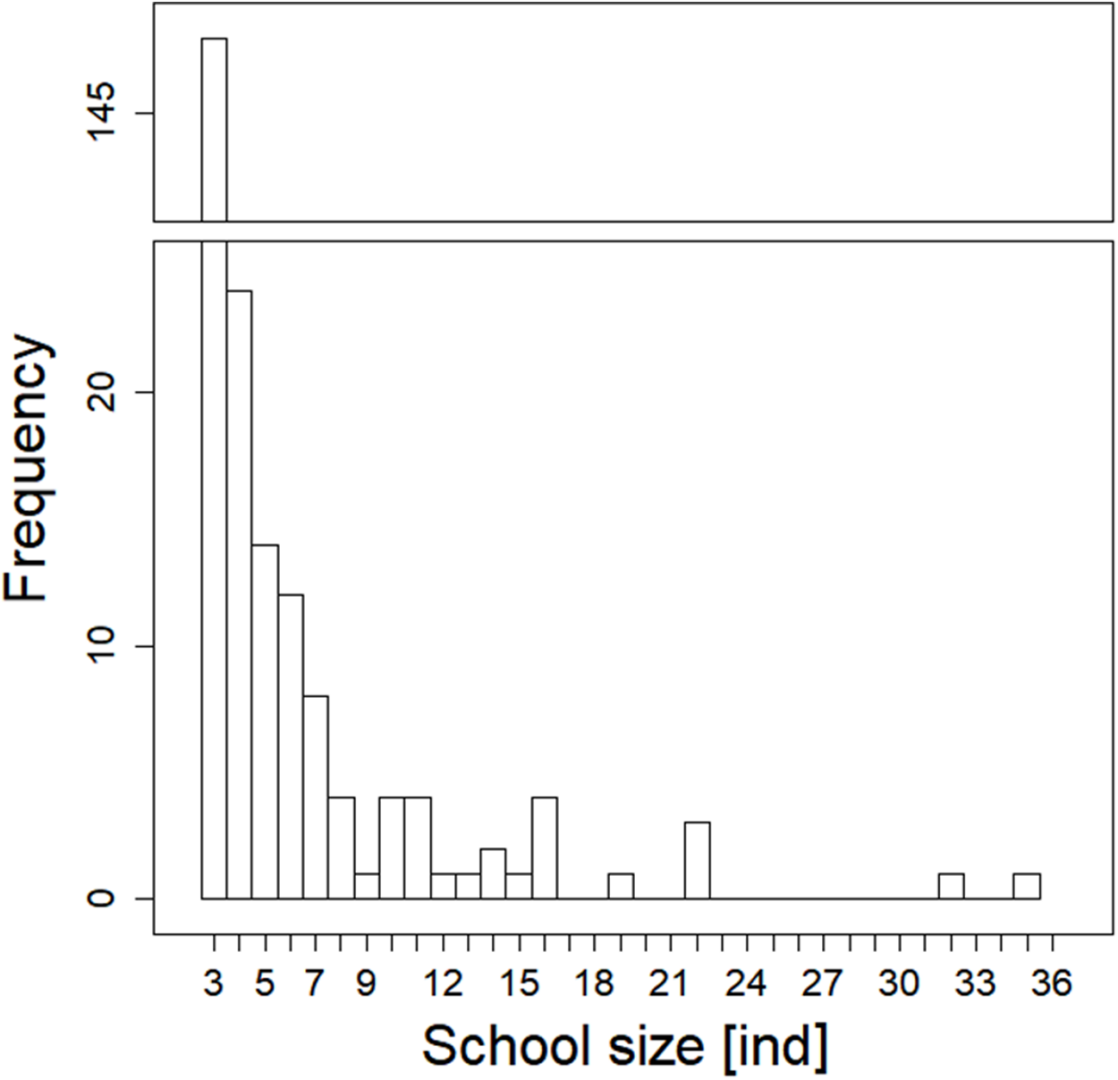
Histogram showing observed frequencies of fish schools of particular size.

During our observation in the epilepagic habitat of the Římov Reservoir a total number of 3,174 fish were captured on video footage. The apparent majority (95%) of the recorded individuals belonged to the species that formed schools, namely freshwater bream, roach, European perch and bleak (Fig. 4). The remaining fish were common carp (*Cyprinus carpio*) and predatory species (3%), specifically asp, Wels catfish, Northern pike and pike-perch. School size ranged from three to 36 members. Smaller school sizes prevailed in the recordings (see Fig. 5) with the average school size 5.6 ± 84% (mean ± SD) individuals. Schools were often composed of more than one species; therefore, the analysis not differentiating species was performed.

**Figure 6:**
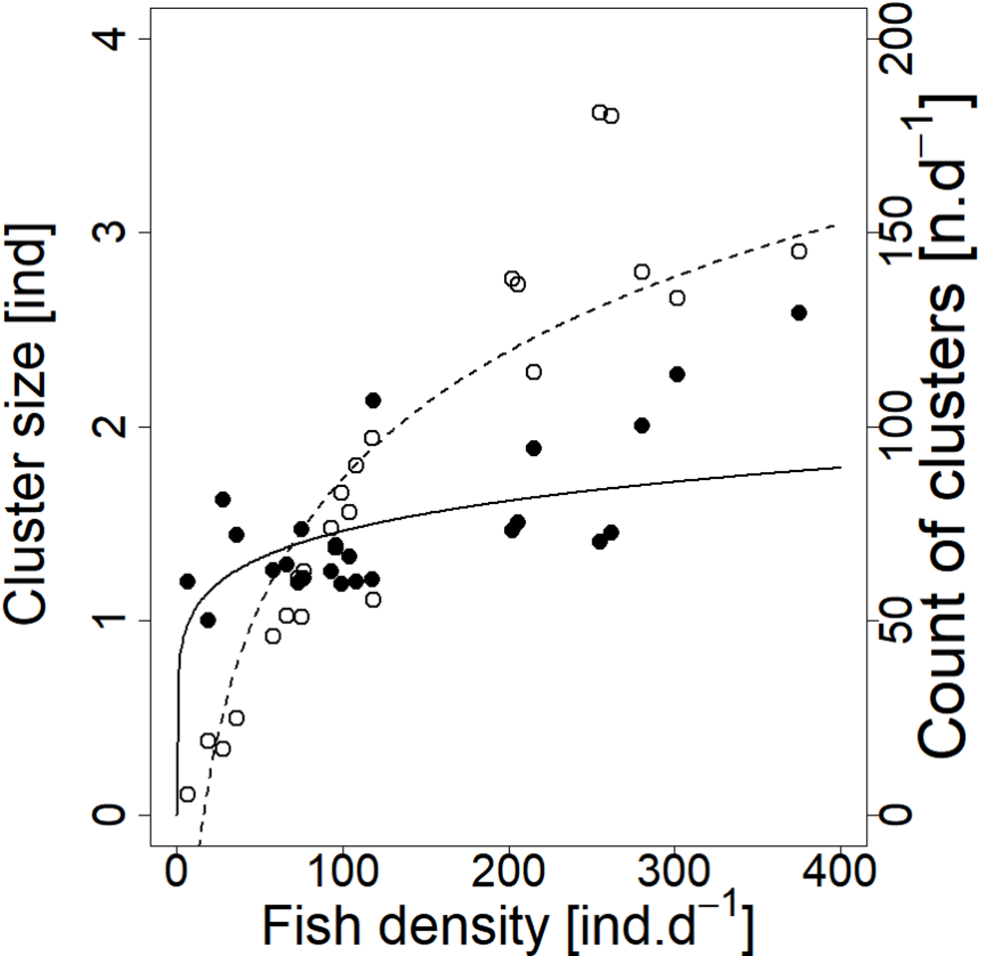
Relationship between cluster count (sum value per day) and cluster size (average diel value) and fish density (sum value per day). Counts of clusters—empty circle and dashed line; cluster sizes—full circle and solid line. For visualisation of log-log model of cluster size and fish density relationship, the predicted *y* value was back exponentiated.

**Figure 7:**
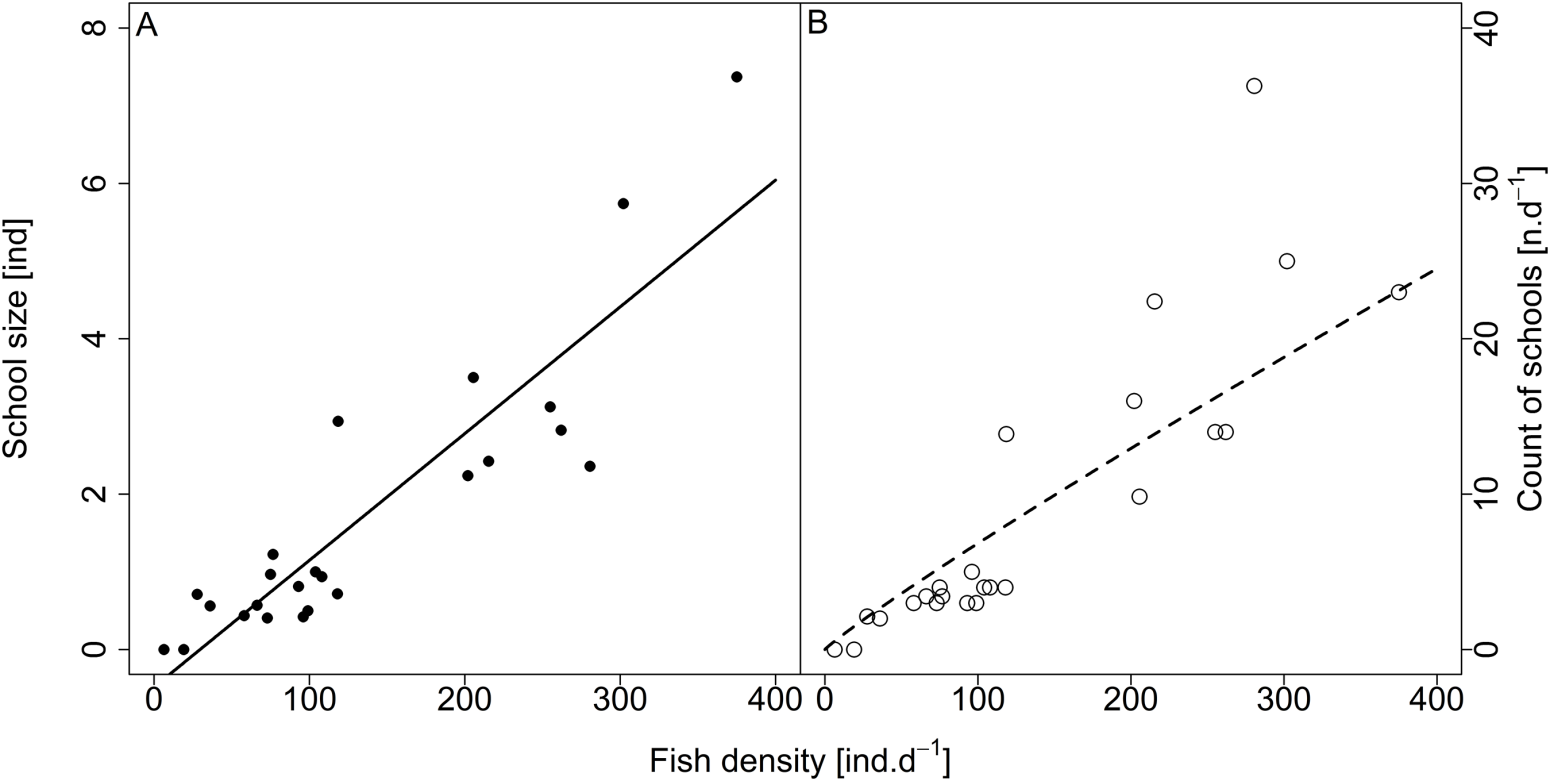
Relationship between (A) school size (average diel value) and (B) count of schools and fish density (sum values per day). Count of schools—full circle and solid line, school size—empty circle and dashed line. For visualisation of log-log model count of schools and fish density relationship, the predicted *y* value was back exponentiated.

**Figure 8:**
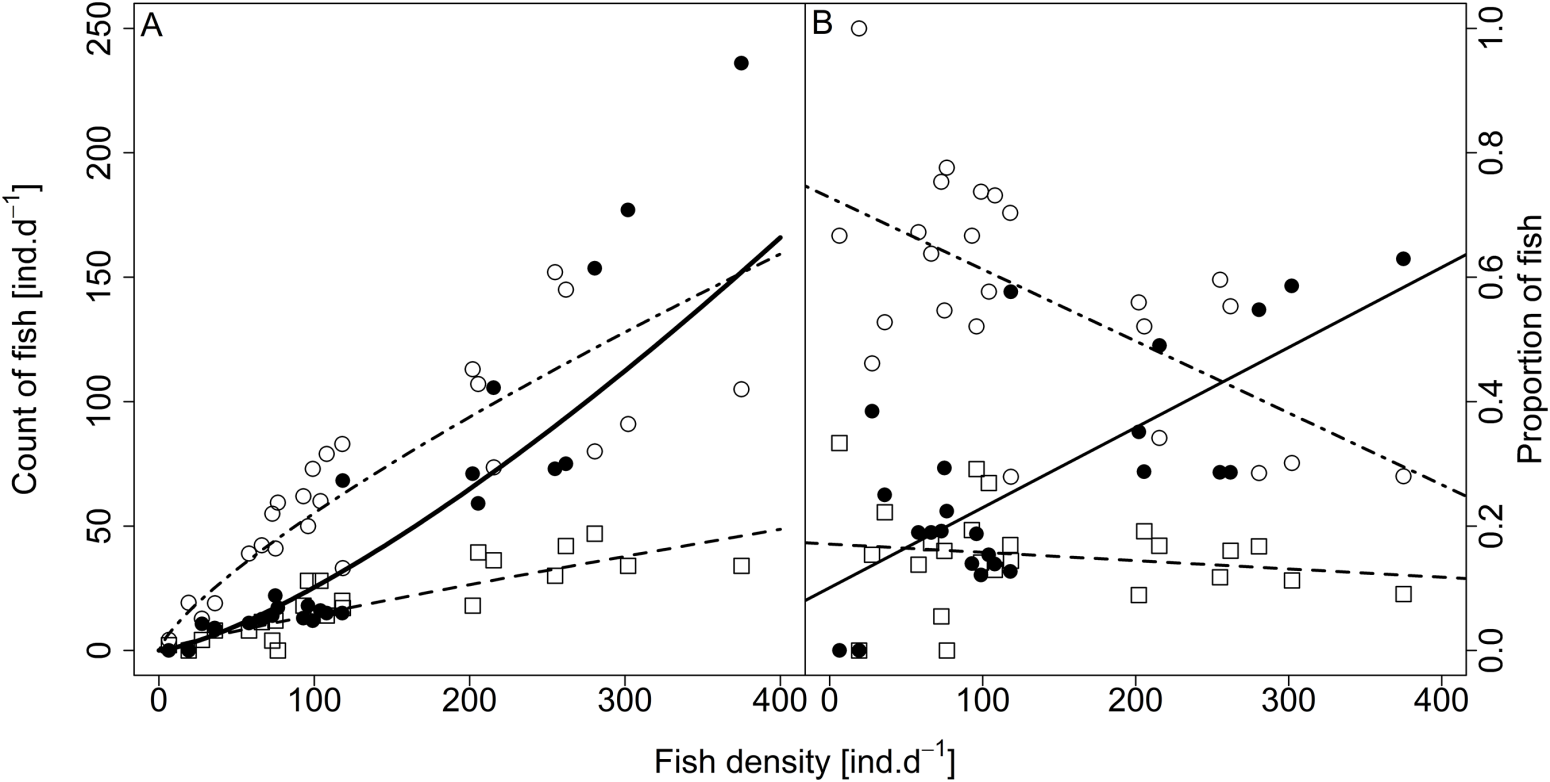
Relationship between (A) counts and (B) proportions of fish in size category (singletons, pairs and schools) and fish density. Singletons—empty circle and dotdash line, pairs—square and dashed line, and schooling individuals—full circle and solid line. For visualisation of log-log model of count of fish in categories (singletons, pairs and schools) and fish density relationship, the predicted *y* value was back exponentiated.

**Figure 9:**
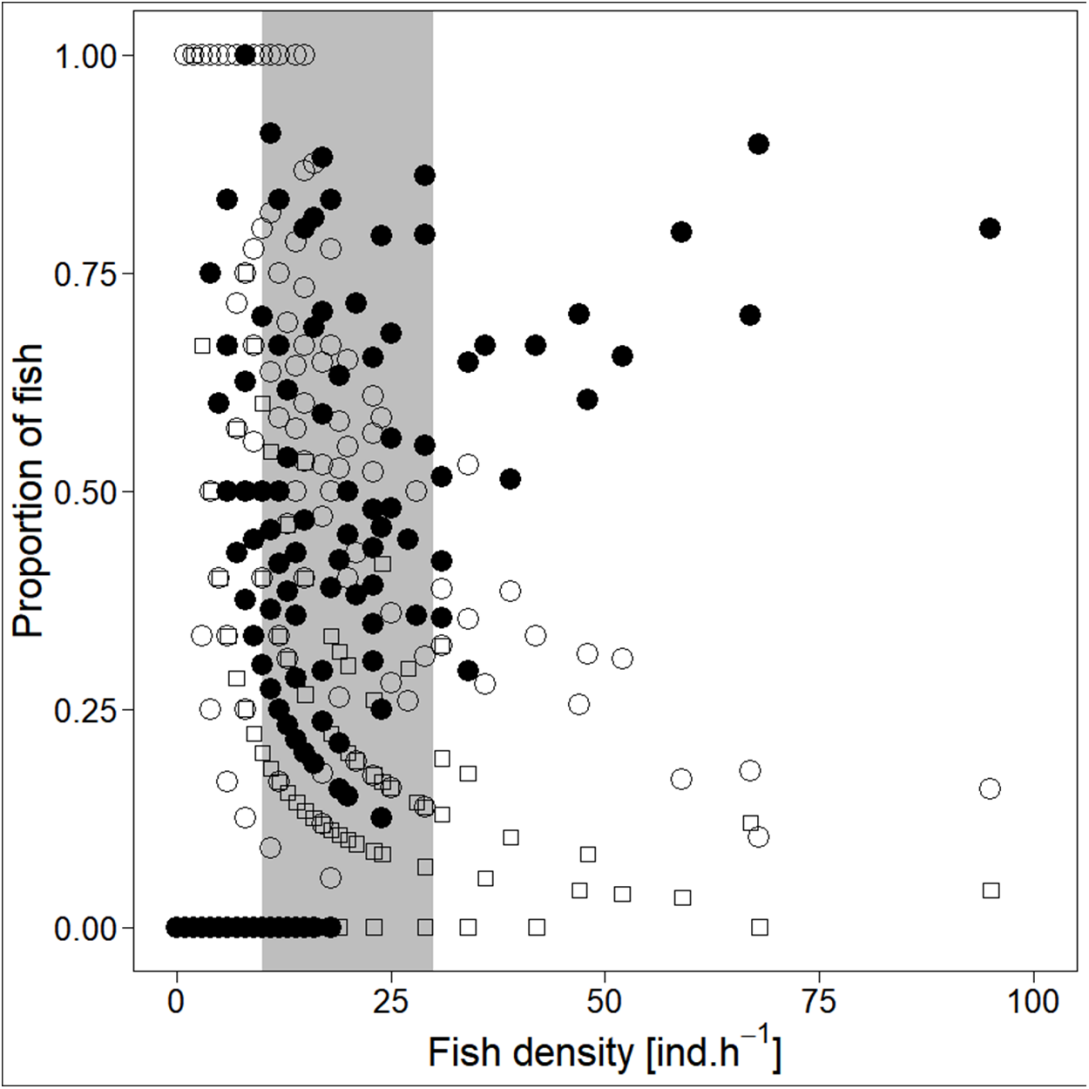
Demonstration of ‘critical density’ on relationship between proportions of fish in size category (singletons, pairs and schools; hourly values) and fish density. ‘Critical density’—depicted by grey area; singletons—empty circle, pairs—square and schooling individuals—full circle.

Analysed fish were organised in 2,183 clusters (comprising singletons, pairs and schools) that included 238 schools (polarised groups of three and more fish). Counts of clusters as a measure of aggregative behaviour observed in the epipelagic habitat showed a slowing increase with fish density (*R*^2^_adj_ = 0.796, *F*_1,22_ = 90.75, *p* < 0.0001; Fig. 6; Table 1), as well as cluster size (cluster size: *R*^2^_adj_ = 0.316, *F*_1,22_ = 11.62, *p* = 0.0025; Fig. 6; Table 1). School size and counts of schools increased linearly with observed fish density (school size: *R*^2^_adj_ = 0.795, *F*_1,22_ = 90.080, *p* < 0.0001; Fig. 7A; counts of schools: *R*^2^_adj_ = 0.840, *F*_1,22_ = 1,201.800, *p* < 0.0001; Fig. 7B; Table 1). Count of singletons showed slowing increase trend with fish density (*R*^2^_adj_ = 0.867, *F*_1,22_ = 144.2, *p* < 0.0001; Fig. 8A; Table 1), whereas count of fish in schools increased rather exponentially (*R*^2^_adj_ = 0.868, *F*_1,22_ = 152.1, *p* < 0.0001; Fig. 8A; Table 1) and count of fish forming pairs increased linearly with small slope (*R*^2^_adj_ = 0.585, *F*_1,22_ = 33.42, *p* < 0.0001; Fig. 8A; Table 1). The proportion of schooling fish linearly increased with fish density (*R*^2^_adj_ = 0.505, *F*_1,22_ = 24.46, *p* < 0.0001; Fig. 8B; Table 1), simultaneously, the proportion of singletons linearly declined (*R*^2^_adj_ = 0.564, *F*_1,22_ = 30.740, *p* < 0.0001; Fig. 8B; Table 1). The proportion of pairs, transitions between singletons and schools, showed no significant relationship with fish density (*p* > 0.05; Fig. 8B; Table 1) and rarely excessed the proportion of 0.2 per day. The data also shows that between densities of 10 and 30 individuals per hour the proportion of fish in schools exceeded the proportion of single fish (Fig. 9). This emergence of schooling behaviour confirms the existence of a threshold in density that stirs fish to group formation called ‘critical density’. Our results prove that fish tend to form schools after reaching a ‘critical density’, which triggers the tendency to join other individuals and form schools whereas tendency to stay alone decreases (as apparent from proportions in size categories; Fig. 8). A slowing increase in count of clusters (Fig. 6) suggests that cluster density might reach an upper limitation resulting in stabilised cluster counts with simultaneous increase in school sizes. This means that although increasing in the density, the encounter rate of schooling fish can stabilise thanks to creating larger schools.

**Table 1:** Best-fit regression models on fish schooling attributes and fish density.

Regression results										
Dependent variable										
	Count of clusters	Cluster size (log(*x*))	Count of schools (log(*x*+1))	School size	Singletons (log(*x*+1))	Fish in pairs (log(*x*+1))	Fish in schools (log(*x*+1))	Proportion of singletons	Proportion of paired fish	Proportion of schooling fish
Fish density (log)	47.227*** (4.957)	0.145*** (0.042)	0.921*** (0.083)		0.765*** (0.064)	0.881*** (0.152)	1.354*** (0.110)			
Fish density				0.016*** (0.002)				−0.001*** (0.0003)	−0.0001 (0.0002)	0.001*** (0.0003)
Constant	−130.669*** (23.168)	−0.286 (0.198)	−2.321*** (0.390)	−0.482 (0.289)	0.490 (0.298)	−1.392 (0.712)	−3.004*** (0.513)	0.728*** (0.050)	0.171*** (0.028)	0.101** (0.044)
Observations	24	24	24	24	24	24	24	24	24	24
*R*^2^	0.805	0.346	0.847	0.804	0.868	0.603	0.874	0.408	0.029	0.527
Adjusted *R*^2^	0.796	0.316	0.840	0.795	0.862	0.585	0.868	0.381	−0.015	0.505
*F* Statistic (d*f* = 1; 22)	90.753***	11.624***	121.830***	90.084***	144.173***	33.419***	152.126***	15.137***	0.658	24.464***

**Notes:**

** *p* < 0.05.

*** *p* < 0.01.

## Discussion

Our study explored the effect of fish density on formation of fish schools in the epipelagic habitat of the European temperate reservoir. Species of the pelagic habitat occurred in corresponding densities with previous studies and school forming ones were the most abundant species (Říha, 2012). Schools were comprised mostly of cyprinid species (freshwater bream, roach and bleak) and European perch. All those species are visually oriented zooplanktivores (Lazzaro, 1987; Vašek & Kubečka, 2004) that utilise the pelagic habitat in our study site in a search for food resource–zooplankton (Vašek & Kubečka, 2004). The absence of shelters in the pelagic habitat can enhance the school formation (Magurran & Pitcher, 1983).

Several tendencies depending on fish density in the habitat were recognized in our recent study. Decrease in proportion of singletons went hand in hand with increase in proportion of fish engaged in schools. Accordingly, school sizes followed linearly increasing trend with fish density and count of clusters and cluster size showed slowing increase with fish density. Altogether, the findings confirm the hypothesis that school formation is triggered by the amount of fish present in the habitat of open water. In other words, schooling behaviour emerged at ‘critical density’ (between 10 and 30 individuals per hour), just the same as was proposed for marine populations (Makris et al., 2009; Maury, 2017). Slowing increase trend of the cluster counts together with increasing amount of schooling fish as well as school and cluster sizes suggest that clusters (observed units of fish) maintain minimal distances from each other. For fish as prey it is disadvantageous to be frequent because predators are able to remember common prey appearance and focus on them (search image; Krebs, 1978). Formation of schools ensures the scattered distribution and evasion of predators as well as faster location of food resource. In addition, the optical properties of the water makes it difficult to recognize friend to foe on long distance and it is only logical that fish encountering same or sympatric species would stick together due to dilution effect of the group (Pitcher & Parrish, 1993). A slowing increase in counts of clusters also corresponds with work of Okubo (1986) who noted that group size and group count tend to be constant. This distribution pattern could serve for limitation of the predator encounter by making themselves rare (Vine, 1971).

This study as well as others on fish (Hensor et al., 2005; Maury, 2017) and other gregarious animals (Wirtz & Lörscher, 1983; Vander Wal, Van Beest & Brook, 2013), confirmed that the key factor affecting the group size is population density. Size of observed schools reached 10 of individuals, with small schools being more frequent than larger ones which is in accordance with other studies (Seghers, 1981; Niwa, 1998; more examples from other taxa in Okubo, 1986). Freshwater school sizes are noticeably smaller than marine schools that can go to thousands of individuals. Smaller densities results in lower number of potential schoolmates. On the other hand, a higher encounter rate in freshwater than in ocean environment (compare Hoare & Krause, 2003; Misund et al., 1998), together with more heterogenous environment and possibilities to migrate to shallow areas, could be the cause for more frequent merging and splitting of freshwater schools. Very large schools are not only exposed to higher competition for resources (Bertram, 1978), but also higher conspicuousness to predators (Turner & Pitcher, 1986) and susceptibility to disease (reviewed in Maury, 2017) and parasites infections (reviewed in Mikheev, 2009), resulting in higher mortality. Nevertheless, the tendency of animals to form large groups with increasing density is undeniable. From opposite point of view, this fact might present an evolutionary mechanism to regulate the population sizes of sympatric species to maintain the equilibrium of ecosystems (Maury, 2017). Some of freshwater bodies suffer from activities of recreational anglers that focused usually on predatory species (Scharf, 2007) which influence could be deeply underestimated (Arlinghaus, Mehner & Cowx, 2002; Lewin, Arlinghaus & Mehner, 2006). Low proportions of piscivorous fish effect ecological interactions and ecosystem structure (Goeden, 1982) even by enhancing the competition ability of gregarious species. This could lead to increase in system trophy, which is undesired for example in water bodies used as drinking water supplies such as Římov Reservoir.

## Conclusions

Our results provide further evidence that the density of fish in the habitat triggers the schooling behaviour. Schooling is in the temperate climate of European water bodies broadly utilised by cyprinid species that dominate the freshwater systems in later succession phases. The question arises as to whether schooling behaviour might be the reason behind the selective advantage responsible for cyprinid dominance in the later succession phases of water bodies. This highlights the need for more ecologically complex studies including the behavioural attributes of specific organisms since they are important for a correct understanding of predator–prey interactions that drives the structure of aquatic ecosystems. Moreover, knowledge of species-specific distribution patterns and aggregative tendencies is crucial for sampling gear selection in attempts to establish the true picture of fish communities.

## Supplemental Information

10.7717/peerj.6378/supp-1 Supplemental Information 1 Density dependance on schooling attributes data.

## Additional Information and Declarations

### Field Study Permissions

The following information was supplied relating to field study approvals (i.e. approving body and any reference numbers): Researchers from the Institute of Hydrobiology were allowed to enter Římov Reservoir with a permit from the Vltava River authority, contract number 300/7225.

### Data Availability

The following information was supplied regarding data availability: The raw measurements are available in the Supplementary File.
